# Evidence Gaps in Clinical Trials of Pharmacologic Treatment for H1-Antihistamine-Refractory Chronic Spontaneous Urticaria: A Systematic Review and Future Perspectives

**DOI:** 10.3390/ph15101246

**Published:** 2022-10-10

**Authors:** Surapon Nochaiwong, Mati Chuamanochan, Chidchanok Ruengorn, Kednapa Thavorn

**Affiliations:** 1 Department of Pharmaceutical Care, Faculty of Pharmacy, Chiang Mai University, Chiang Mai 50200, Thailand; 2 Pharmacoepidemiology and Statistics Research Center (PESRC), Chiang Mai University, Chiang Mai 50200, Thailand; 3 Division of Dermatology, Department of Internal Medicine, Faculty of Medicine, Chiang Mai University, Chiang Mai 50200, Thailand; 4 Ottawa Hospital Research Institute, Ottawa Hospital, Ottawa, ON K1H 8L6, Canada; 5 School of Epidemiology and Public Health, Faculty of Medicine, University of Ottawa, Ottawa, ON K1G 5Z3, Canada

**Keywords:** biologic agents, chronic spontaneous urticaria, clinical trials, core outcomes sets, evidence gaps, H1-antihistamine-refractory, minority groups

## Abstract

No data addressing issues concerning disparities in participant and trial characteristics and trial outcome reporting have been established in clinical trials for H1-antihistamine-refractory chronic spontaneous urticaria (CSU). To better harmonize and compare the different treatment interventions, we systematically evaluated the overall landscape of pharmacological treatments for H1-antihistamine-refractory CSU clinical trials published between 2000 and 2021. This systematic review included 23 randomized clinical trials involving 2480 participants from 22 countries. We found significant increases in the number of globally published and newly tested drugs, especially biologic drugs. Regarding relatively small trials, we found that people living with H1-antihistamine-refractory CSU who were identified as members of minority groups (non-white population), populations of regions other than North America/Europe, and populations of low- to lower/upper-middle-income countries are underrepresented. Most trials were designed to evaluate treatment efficacy and safety profiles; however, less than half of the included trials reported the patient’s perspective in terms of patient-reported outcomes. Disparities in outcome reporting, including clinimetric tools for assessing treatment response and outcome sets, were observed. To close the evidence gap in H1-antihistamine-refractory CSU trials, strategies for improving trial and participant enrollment and standardizing core outcome sets for trial reporting are needed.

## 1. Introduction

Chronic spontaneous urticaria (CSU) is characterized by wheals and itching lasting for more than six weeks, with or without angioedema and with no identifiable trigger [1]. This condition affects patients of all ages and has an estimated global prevalence of 4.4%, which has been increasing over time [2]. Despite the widespread use of licensed doses and up-dosing (i.e., two- to four-fold higher than approved doses) of H1-antihistamines, less than half of patients with CSU responded adequately to this first-line therapy [1,3]. Furthermore, CSU can severely impact health-related quality of life (HRQOL), reduce physical and social interactions, affect work or school performance, and negatively affect mental health and psychosocial issues, particularly refractory cases [1,4].

Over the past decade, therapy for people living with CSU, mainly those unresponsive to licensed doses and up-dosing of H1-antihistamines, has evolved rapidly [5,6,7]. Currently, randomized trials of novel therapies to offer pharmacological treatment options for the management of H1-antihistamine-refractory CSU are ongoing [1,5]. These promising pharmacological therapies possess biological, immunosuppressive, and other pharmacological qualities. Recent evidence has suggested that biologic agents such as ligelizumab (72 or 240 mg) and omalizumab (300 or 600 mg) appear to be effective treatments (moderate to large beneficial effect) and were closely associated with improved HRQOL in people living with H1-antihistamine-refractory CSU [8,9].

To date, there is a need to improve disparities in the diversity of participant enrollment based on equity and representativeness to close the gap in dermatologic trials [10]. According to the United States Food and Drug Administration (FDA), in 2014, an action plan was recognized and developed to support and encourage diversity in randomized clinical trials so that more concise information regarding the representativeness of participants in trials can be published [11]. Theoretically, effective treatments may limit generalizability or effectiveness for all populations when diverse populations are lacking or underrepresented in clinical trials [12]. Recently, a standardized set of outcomes to capture and assess treatment intervention effectiveness and safety profiles has been brought to the scientific community’s attention.

To our knowledge, no data addressing issues concerning disparities in participant and trial characteristics and trial outcome reporting have been established in clinical trials for H1-antihistamine-refractory CSU. To better harmonize and compare the different treatment interventions, we performed a systematic review to assess the evidence gap, consistency in outcome reporting, and representation across global pharmacological treatment clinical trials involving people living with H1-antihistamine-refractory CSU.

## 2. Methods

### 2.1. Protocol and Literature Search

The pre-specified protocol and living systematic review update was registered on the International Prospective Register of Systematic Reviews (PROSPERO, CRD42020196592). However, the pre-specified protocol was amended to focus on randomized clinical trials owing to the limited availability of relevant data on comparative effectiveness observational studies. Therefore, we decided not to include non-randomized studies in the present study. This systematic review followed the Preferred Reporting Items for Systematic Reviews and Meta-Analyses 2020 statement [13]. Due to the nature of the systematic review and meta-analysis, ethical approval was not required.

Regarding the pre-specified protocol, with the help of an experienced medical librarian, we searched seven electronic databases including Medline, Embase, PubMed, Cochrane Library, Web of Science, Scopus, and CINAHL. We also searched grey literature on Google Scholar, clinical trial registers, and preprints reports. We constructed the search strategy based on the combinations of main keywords or medical subject headings regarding CSU (e.g., “Chronic spontaneous urticaria” OR “Chronic idiopathic urticaria” OR “Refractory urticaria” OR “Hives”). Moreover, search terms in relation to types of interventions were browsed according to the individual drugs or their pharmacological classes (e.g., “Anti-immunoglobulin E” OR “Monoclonal antibody” OR “Bruton tyrosine kinase inhibitor” OR “CRTH2 antagonist” OR “IL-1 inhibitor” OR “anti-tumor necrosis factor alpha” OR “Immunosuppressive Agent” OR “Calcineurin inhibitor”).

A comprehensive literature search was performed from the inception of each database until 19 April 2021, with no language restrictions. Potential trials were also supplemented by searching the reference lists of included studies, previous systematic reviews, and major international scientific conference meetings (dermatology, allergy, and immunology congress). The search strategy details for each database are available in Supplementary Tables S1 and S2.

### 2.2. Article Screening

Two investigators (SN and MC) independently screened the eligible titles and abstracts of articles identified by the systematic search. Subsequently, potentially relevant full-text articles were reviewed against the study inclusion/exclusion criteria for the final set of included studies. Potential non-English-language eligible studies were translated before the full-text appraisal. Any disagreement was resolved through team discussion.

The inclusion and exclusion criteria details were described previously (Supplementary Table S3) [8,9]. In brief, we included randomized clinical trials (parallel and cross-over trials) that (i) involved adolescents or adults (12 years or older) who were diagnosed with CSU refractory to H1-antihistamines (standard dose or up-dosing) and (ii) used validated measurement tools for urticaria treatment response assessment with a follow-up period of two weeks onward. For companion trials with overlapping participants and study periods, relevant information was assembled from the study that provided the most detailed information.

However, we excluded clinical trials that investigated body weight-based or immunoglobulin E level-based dosing of omalizumab, as early studies among patients with CSU showed no benefit of this approach [14]. Moreover, trials that used terfenadine up-dosed or combined with other treatments were excluded from this review because this treatment is no longer available in current practice. All placebos across the included trials were defined as standard doses or an up-dosing of H1-antihistamines in conjunction with rescue medications.

### 2.3. Data Extraction

After screening articles against the inclusion and exclusion criteria, data were collected via full-text abstraction of the included clinical trials. Two investigators (SN and MC) independently extracted pre-specified information using a standardized approach. Pre-specified data extraction was managed via a Microsoft Excel spreadsheet (data extraction details are described in Table 1). The categorization of race and ethnicity was based on the National Institutes of Health—United States Office of Budget and Management, Revisions to the Standards for the Classification of Federal Data on Race and Ethnicity, 2020 [15]. Based on participant-level characteristics, we classified trials that indicated any race other than white as a trial involving a non-white population. To establish the underrepresentation of race and ethnicity across the included clinical trials, the cutoff value (<20%) that reflects the United States census was employed [10]. Discrepancies and uncertainties were resolved through discussion until a consensus was reached. Two investigators (CR and KT) independently cross-checked the final dataset.

### 2.4. Risk-of-Bias Assessment

Two investigators (SN and MC) independently and critically appraised the methodological quality of each included trial using the Cochrane revised tool for risk of bias assessment (RoB 2) [16]. Cochrane RoB 2 consists of six bias domains, including the randomization process, deviations from intended interventions, missing outcome data, measurement of the outcome, and selection of the reported result. The overall risk of bias of the included trials was then classified as low, some concerns, or high risk of bias [16].

### 2.5. Statistical Analysis

Descriptive statistics were used and are expressed as frequency and percentage, mean ± standard deviation, or median with a range (min–max), as appropriate. To explore the disparities and evaluate the evidence gap across continents and the capacity to conduct clinical trials, the included trials were classified as high-income or lower/upper-middle-income country trials based on the World Bank 2021 income grouping. Statistical significance of differences in the evidence gap in terms of participant and trial characteristics and outcomes of interest across the included trials according to the World Bank income grouping was determined using Fisher’s exact tests. Two-tailed tests with *p*-values < 0.05 were considered statistically significant. All analyses were performed using Stata 14.0 (StataCorp LLC).

## 3. Results and Discussion

### 3.1. Overview of Included Trials for H1-Antihistamine-Refractory CSU

**Figure 1:**
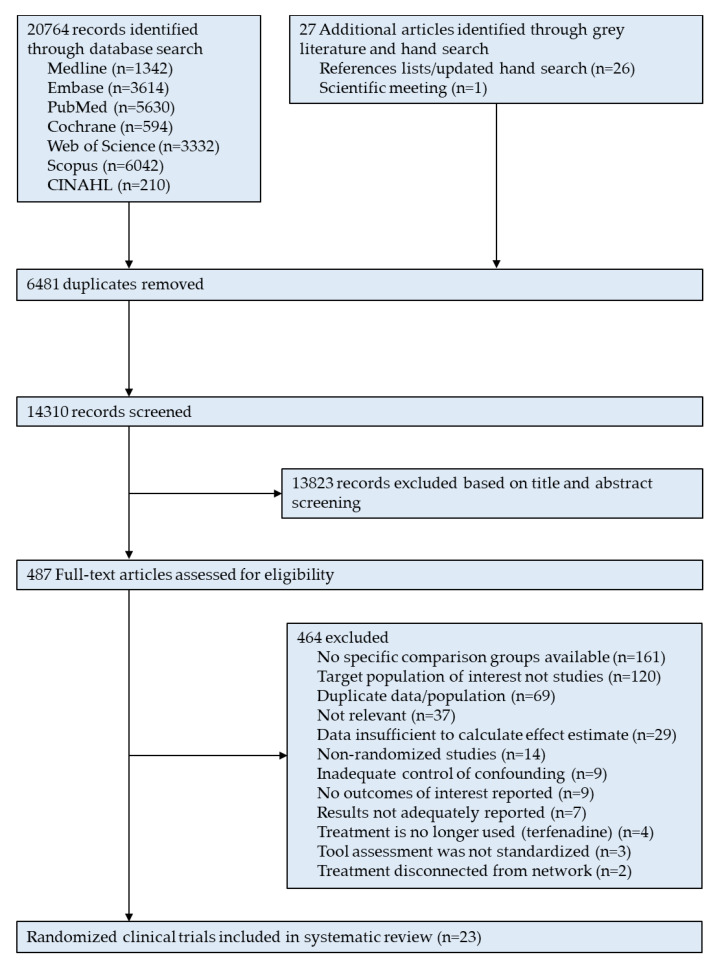
Preferred Reporting Items for Systematic Reviews and Meta-Analyses (PRISMA) flow diagram of trials included in the systematic review.

Based on a systematic search approach, 20,796 records were identified. After deduplication and screening based on title and abstract, we identified 492 articles for full-text review. Of these, 23 randomized clinical trials (21 parallel-group [14,17,18,19,20,21,22,23,24,25,26,27,28,29,30,31,32,33,34,35,36] and two cross-over [37,38] trials) fulfilled the study inclusion and exclusion criteria, and were included in this systematic review (Figure 1).

### 3.2. Participant and Trial Characteristics

The participant and trial characteristics of all the included trials are illustrated in Table 2. The included trials comprised 2480 participants (range, 20–340 participants per trial) from 22 different countries, with a treatment duration of 12.0 ± 7.1 (range, 3.0–24.0) weeks. The reported mean age was 41.2 ± 3.4 years (range, 32.2–45.7; mainly adult participants) and the reported proportion of women was 64.8% (range, 6.2–86.7%). However, 10 (43.5%) trials did not report race or ethnicity. More than one-half of the included trials (13 trials, 56.5%) were conducted in participants who were unresponsive to licensed doses of H1-antihistamine. Regarding trial characteristics, 15 (65.2%) of the included trials were multicenter trials, 21 (91.3%) were placebo-controlled trials, 20 (87.0%) used a double-blind trial design, 18 (78.3%) were two-arm trials, and 13 (56.5%) received industry sponsorship. Of the 23 included trials, 13 (56.5%) had a low risk of bias, 10 (43.5%) had some concerns, and no studies had a high risk of bias.

Twelve (52.2%) of the included trials were conducted in North America or Europe, seven (30.4%) in international trial locations, and four (17.4%) in other locations. Based on the global distribution of pharmacological treatments for H1-antihistamine-refractory CSU, the top two countries that conducted trials were Germany (nine trials) and the United States (eight trials).

**Figure 2:**
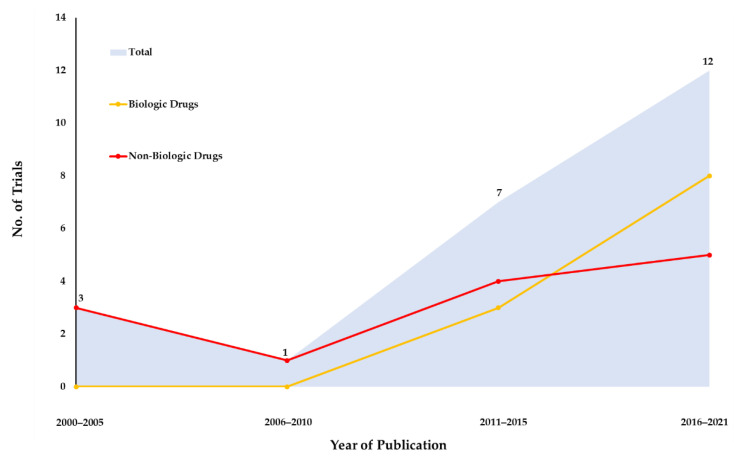
Time trend in the publication of pharmacologic treatments for H1-antihistamine-refractory chronic spontaneous urticaria trials.

The included trials were published between the years 2000 and 2021. The type of pharmacological intervention was identified as biologic drugs in 12 (52.2%) trials, immunosuppressive drugs in five (21.7%), and others in six (26.1%), which investigated 18 different pharmacological interventions or dosages and one placebo (usual care treatment). Notably, in 2015 and 2021, the number of biological drugs used dramatically increased by 300% and 800%, respectively; the number in 2005 was zero (Figure 2). Meanwhile, the number of non-biological drugs investigated in clinical trials annually revealed little change over time.

### 3.3. Trial Outcome Reporting

**Figure 3:**
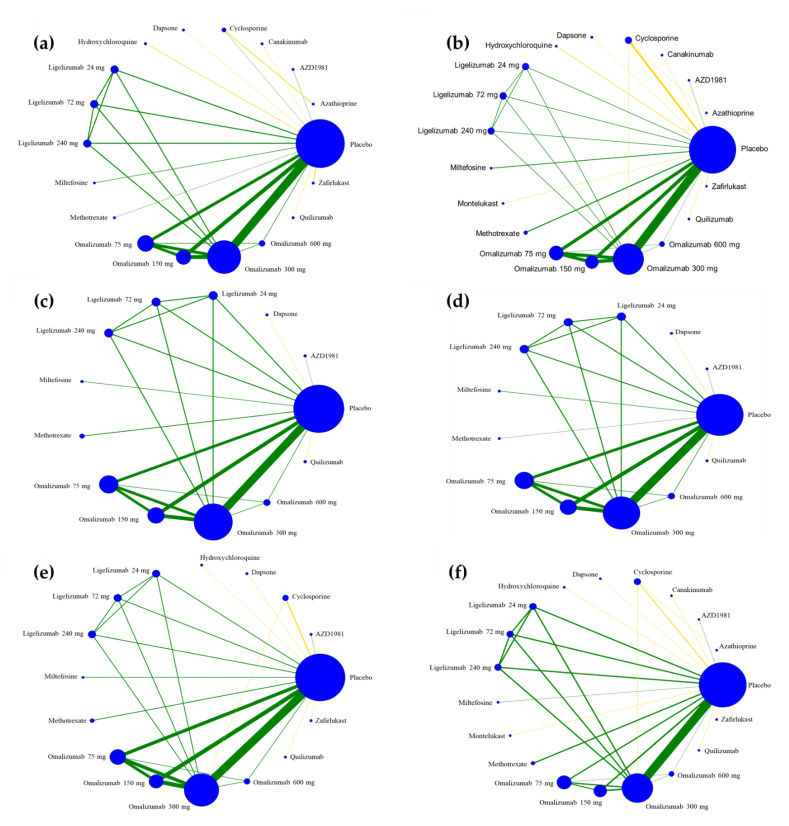
Geometry networks for pharmacologic treatment-level comparisons. (**a**) urticaria symptoms; (**b**), unacceptability of treatment (all-cause study dropout); (**c**) pruritus severity; (**d**) hive severity; (**e**) adverse event; (**f**), serious adverse event. Nodes denote pharmacological treatments and lines denote trials of the corresponding treatment comparison. The size of a node is proportional to the number of trials that included the corresponding treatment. The thickness of the lines corresponds to the number of trials performing each comparison (green and yellow lines represent studies with a low risk of bias and some concerns, respectively, according to the overall risk-of-bias assessment).

The outcomes of interest were categorized into three groups: (i) treatment efficacy, (ii) safety profiles, and (iii) patient-reported outcomes (Table 3). Regarding treatment efficacy, 16 (69.6%) trials reported treatment response for urticaria symptoms using the urticaria activity score over 7 days (UAS7). Meanwhile, 13 (56.5%) and 12 (52.2%) trials reported the component of urticaria symptoms—pruritus and hives severity, respectively. Regarding safety profiles, the unacceptability of treatment (all-cause study dropout), incidence of adverse events, and serious adverse events were the most reported outcome theme (≥80% of the included trials). However, less than half of the included trials recognized patient-reported outcomes. Based on validated tools, patient-reported outcomes included a measure of HRQOL (dermatology-specific, chronic urticaria-specific, angioedema-specific, and generic measures), impact on sleep, and general well-being. Regarding treatment-level comparisons, all possible individual pharmacological nodes for each outcome of interest are illustrated in Figure 3.

### 3.4. Evidence Gap across Included Trials

No clinical trials are available in African and low-income countries. Meanwhile, limited clinical trials have been conducted in regions other than America and Europe. Based on the World Bank income grouping, 18 (78.3%) of the included trials were conducted in high-income countries and five (21.7%) in lower/upper-middle-income countries. The distribution of participants and trial characteristics (Table 2), including age (*p* < 0.001), duration of CSU (*p* = 0.029), trial setting (*p* = 0.033), trial location and continent (*p* = 0.001), the control group in a trial (*p* = 0.040), trial blinding (*p* = 0.006), and funding (*p* = 0.001), demonstrated a statistically significant association with the country income groups (high-income vs. lower/upper-middle-income countries). Moreover, trials in high-income countries were more likely to use a well-established tool—UAS7—and report on the component of urticaria symptoms (severity of pruritus and hives) compared with trials conducted in lower/upper-middle-income countries (all *p* < 0.050, Table 3). However, disparities in reported results, particularly patient-reported outcomes in the core outcome set and measurement tools, were observed across the included trials.

### 3.5. Summary of the Findings

This systematic review highlighted a global evidence gap and provided important information regarding pharmacological treatments for H1-antihistamine-refractory CSU clinical trials. Significant global increases in published clinical trials and new investigational medicinal products, especially biologic drugs, demonstrate the progress made between 2000 and 2021. However, we hypothesized that people living with H1-antihistamine-refractory CSU who are members of minority groups (non-white population), part of regions other than North America/Europe (i.e., Africa, Asia, and South America), and live in low- to lower/upper-middle-income countries would be underrepresented in CSU clinical trials.

Currently, licensed biologic therapy—omalizumab (anti-immunoglobulin E)—has become the frontline treatment and revolutionized the management of H1-antihistamine-refractory CSU [1]. Subsequently, overall increases were observed in the number of H1-antihistamine-refractory CSU drugs investigated. Based on promising mechanisms, there has been a rapid surge of clinical trials among new biologics drugs that reduce mast cell activation by blocking activating pathways and engaging inhibitory receptors or mast cell members [5,6,7].

### 3.6. Comparison with Other Studies

The poor representation of minority groups in this study is consistent with previous systematic reviews [10]. Chen et al. (2022) [39] examined the diversity of participants in dermatologic clinical trials in the United States published conducted between 2010 and 2020 and found that clinical trials that included at least 20% non-white participants were noticeably few. Of note, 10 (43.5%) of the trials included in this study did not report on race or ethnicity. One possible explanation for the poor enrollment of racial/ethnic minority groups in clinical trials is that existing national or international policies for promoting patient engagement in randomized clinical trials are not effective. Currently, the United States FDA has implemented an action plan to support and encourage diversity in randomized clinical trials conducted by the industry to improve and publish transparent data regarding race or ethnicity [11]. However, these FDA regulations focus on investigating new drugs and are not implemented in all industry-sponsored and non-industry-sponsored clinical trials.

Regarding infrastructure and economic indices for clinical trials, only five (21.7%) of 23 trials were conducted in lower/upper-middle-income countries between 2000 and 2021. Moreover, the included trials were generally small-scale, with a median sample size of 75 (range, 20–340) participants and a median study treatment duration of 12 (range, 3–24) weeks. Remarkably, our study illustrated a statistically significantly different in the age of participants, duration of CSU, trial setting, trial location and continent, the control group in a trial, trial blinding, and funding between high-income and lower/upper-middle-income countries. Unfortunately, we found no clinical trials in African regions or low-income countries, suggesting that the pharmaceutical industry and clinical research organizations need to set policies and harmonize trial designs to increase participants’ demographic and geographical diversity.

Regarding outcome reporting, most included trials were designed to evaluate urticaria treatment responses and safety profiles. However, disparities in the clinimetric tools for assessing treatment responses have been observed. More than one-half of the included trials (16 trials, 69.6%) used UAS7—a validated tool suggested by the 2021 joint update guideline recommendations from the European Academy of Allergology and Clinical Immunology, Global Allergy and Asthma European Network, European Dermatology Forum, and the Asia Pacific Association of Allergy, Asthma and Clinical Immunology [1]; other trials used other clinimetric tools, including daily UAS, urticaria severity score (USS), or visual analog scale (VAS, scale 0–100). Beyond treatment efficacy and safety profiles, less than half of the included trials reported patients’ perspectives regarding patient-reported outcomes. Most trials assessed HRQOL, but lacked information regarding other aspects of patient-reported outcomes, such as sleep problems, symptom burden, psychosocial and mental health problems, and work or school impairment. Although the Cochrane Skin—Core Outcome Set Initiative has been developed as a core outcome set for several skin conditions, there is a lack of specific outcome sets for patients with CSU [40]. To better compare across-trial results, we recommend that standardized reporting in core outcome sets for people living with CSU (including efficacy, safety events, and patient-reported outcomes) be developed and applied to make trial evidence more useful [41].

### 3.7. Strengths and Limitations

To the best of our knowledge, this is the first systematic review to elucidate the disparities and evidence gaps across pharmacologic treatment clinical trials for H1-antihistamine-refractory CSU. However, our systematic review has some limitations. First, age was reported in various ways—by category, mean, range, and percentage older than 12 years, with the exclusion age criteria (i.e., 65, 70, or 75 years)—resulting in difficulty in addressing age disparities. Second, we could not assess representativeness based on sexual identity and orientation because of the lack of information on these issues. Third, 10 (43.5%) trials did not report race or ethnicity; therefore, we had to deduce racial makeup based on the country or judge that neither race nor ethnicity was reported owing to insufficient data. Lastly, we only assessed published H1-antihistamine-refractory CSU randomized clinical trials that used validated measurement tools for outcomes assessment. As a result, we lack information on ongoing studies based on clinical trial registries, non-randomized studies, and post-marketing trials, as well as other aspects of dermatology trials.

### 3.8. Implications for Conducting Clinical Trials, Future Research, and Conclusion

Establishing disparities and evidence gaps in H1-antihistamine-refractory CSU clinical trials is the first step toward developing a system for conducting clinical trials that involve an increasingly diverse population. Given that the clinical trials included in this systematic review were generally small, we postulate that our findings are informative for the pharmaceutical industry, researchers, patients, and policymakers to promote medical innovation in CSU. Collectively, we advocate the inclusion of diverse populations in CSU trials globally. Different study site selections in industry-sponsored clinical trials based on multinational studies are required to improve diversity in participant enrollment and drug development for diverse populations. Besides population availability and timely recruitment, patient enrollment based on heterogonous inclusion of age, sex, and race or ethnicity should be employed in the protocol generation phase. Ultimately, to conduct clinical trials, experience is not required; the pharmaceutical industry and clinical research organizations should collaborate with inexperienced trial sites, particularly in low- or lower/upper-middle-income countries, if they have access to the relevant patient population.

To facilitate and encourage the formation of a highly motivating environment for pharmaceutical innovation, it is essential for future research and development strategies to create equitable access to new investigational medical products and the efficiency of pharmaceutical research in high- and lower/upper-middle-income countries. Furthermore, apart from international collaborative clinical trials, the approaches being considered at present will need head-to-head trials with high methodological quality and harmonized trial design and outcomes to help inform subsequent international guidelines for managing people living with CSU.

## 4. Conclusions

In this systematic review, the number of randomized clinical trials for the pharmacologic treatment of H1-antihistamine-refractory CSU increased between 2000 and 2021, particularly in biologic drug intervention trials. However, relatively small trial sizes, underrepresentation of minority groups (non-white populations, populations of regions other than North America/Europe, and low- to lower/upper-middle-income countries), and disparities in outcome reporting were observed across H1-antihistamine-refractory CSU clinical trials. To close the evidence gap in H1-antihistamine-refractory CSU clinical trials, strategies for improving clinical trials and participant enrollment and standardizing core outcome sets for trial reporting are needed.

## Figures and Tables

**Table 1:** Pre-specified data extraction.

Domain	Detail
Participant characteristics	Number of participants enrolled Mean or median age of study participants and age inclusion criteria Reported proportion of females Race or ethnicity reporting based on National Institutes of Health—United States Office of Budget and Management, 2020 Definition of H1-antihistamine-refractory CSU: refractory to (i) licensed-dose antihistamines, (ii) up-dosing antihistamines (two- to four-fold the licensed-dose), (iii) mixed/not specified Duration of CSU Pharmacologic intervention class: biologic drugs, immunosuppressive drugs, and others
Trial characteristics	Year of publication Trial setting: monocentric vs. multicenter Trial location: North America/Europe, others, and international Trial design: parallel-group vs crossover design Control group in trial: placebo-controlled vs. active-controlled trial Trial blinding: open-label, single-blind, double-blind, triple-blind, or quadruple-blind Number of arms in trial Study treatment period in week Funding: industry sponsorship, partial industry sponsorship, academic/government, none, or not reported Overall risk of bias based on the Cochrane revised tool for risk-of-bias assessment (RoB2): low, some concerns, or high-risk of bias
Outcomes of interest	Measurement tools and definition of outcomes Treatment efficacy: urticaria symptom, pruritus severity, and hive severity Safety profiles: all-cause study dropout, incidence of adverse events, incidence of serious adverse events Patient-reported outcomes: HRQOL (i.e., dermatology-specific, chronic urticaria-specific, angioedema-specific, or generic measure), impact on sleep, treatment satisfaction, or others mental health and psychosocial issues

Abbreviations: CSU, chronic spontaneous urticaria; HRQOL, health-related quality of life.

**Table 2:** Characteristics of included trials.

Participant and Trial Characteristics	Number of Trials (%)			*p*-Value
	Overall (*n* = 23)	High-Income Countries (*n* = 18) ^†^	Lower/Upper-Middle-Income Countries (*n* = 5) ^†^	
Total enrollment, participants; median (range, min–max)	2480; 75 (20–340)	2251; 83 (20–340)	229; 35 (29–80)	0.279
<50 participants	10 (43.5)	7 (38.9)	3 (60.0)	0.563
50–100 participant	8 (34.8)	6 (33.3)	2 (40.0)	
>100 participants	5 (21.7)	5 (27.8)	0 (0.0)	
Reported mean age in year, grand mean ± S.D.; Median (range, min–max); missing data for one trial (4.3%)	41.2 ± 3.4; 42.5 (32.2–45.7)	41.3 ± 1.8; 42.7 (38.7–45.7)	36.2 ± 4.7; 34.9 (32.2–42.8)	<0.001
Age inclusion				
Only adults included	16 (69.6)	12 (66.7)	4 (80.0)	1.000
Mixed children and adolescents/adults included	7 (30.4)	6 (33.3)	1 (20.0)	
Reported % female, grand mean ± S.D.; Median (range, min– max); missing data for one trial (4.3%)	64.8 ± 19.2; 69.6 (6.2–86.7)	63.9 ± 20.6; 70.6 (6.2–86.7)	68.5 ± 12.4; 65.0 (58.6–85.4)	0.677
Race/ethnicity reporting				
>80% white representation	9 (39.1)	9 (50.0)	0 (0.0)	0.108
≥20% non-white representation	4 (17.4)	3 (16.7)	1 (20.0)	
Neither race nor ethnicity reported	10 (43.5)	6 (33.3)	4 (80.0)	
Type of refractory CSU				
Refractory to licensed-dose antihistamines	9 (39.1)	9 (50.0)	0 (0.0)	0.108
Refractory to up-dosing antihistamines (two- to four-fold the licensed-dose)	10 (43.5)	6 (33.3)	4 (80.0)	
Mixed/not specified	4 (17.4)	3 (16.7)	1 (20.0)	
Duration of CSU in year, grand mean ± S.D.; Median (range, min–max); missing data for six trials (26.1%)	5.0 ± 2.8; 5.1 (0.5–11.5)	5.9 ± 2.4; 5.9 (2.4–11.5)	2.4 ± 2.4; 1.7 (0.5–5.9)	0.029
Pharmacologic intervention class of studies: Specific intervention (no. of trials) ^‡^				
Biologic drugs: Canakinumab (1), Ligelizumab (1), Omalizumab (10), Quilizumab (1)	12 (52.2)	11 (61.1)	1 (20.0)	0.214
Immunosuppressive drugs: Azathioprine (1), Cyclosporine (4), Methotrexate (2)	5 (21.7)	3 (16.7)	2 (40.0)	
Others: AZD1981—CRTh2 antagonist (1), Dapsone (1), Hydroxychloroquine (1), Miltefosine (1), Montelukast (1), Zafirlukast (1)	6 (26.1)	4 (22.2)	2 (40.0)	
Year of publication				
Before 2015	10 (43.5)	8 (44.4)	2 (40.0)	1.000
2015–2021	13 (56.5)	10 (55.6)	3 (60.0)	
Trial setting				
Monocentric	8 (34.8)	4 (22.2)	4 (80.0)	0.033
Multicenter	15 (65.2)	14 (77.8)	1 (20.0)	
Trial location: Country (no. of trials)				
North America/Europe: France (1), Germany (3), Italy (1), Switzerland (2), Türkiye (1), United Kingdom (1), United States (3)	12 (52.2)	11 (61.1)	1 (20.0)	0.001
Others: Colombia (1), India (2), Thailand (1)	4 (17.4)	0 (0.0)	4 (80.0)	
International (includes Australia, Canada, Denmark, France, Germany, Greece, Italy, Japan, Korea, New Zealand, Poland, Russian Federation, Singapore, Spain, Taiwan, Türkiye, United Kingdom, United States)	7 (30.4)	7 (38.9)	0 (0.0)	
Trial Design				
Parallel-group	21 (91.3)	17 (94.4)	4 (80.0)	0.395
Crossover	2 (8.7)	1 (5.6)	1 (20.0)	
Control group in trial				
Placebo-controlled trial	21 (91.3)	18 (100.0)	3 (60.0)	0.040
Active-controlled trial	2 (8.7)	0 (0.0)	2 (40.0)	
Trial Blinding				
Open-label/single-blind	3 (13.0)	0 (0.0)	3 (60.0)	0.006
Double-blind	20 (87.0)	10 (100.0)	2 (40.0)	
Arms in trial				
2	18 (78.3)	13 (72.2)	5 (100.0)	0.545
≥3	5 (21.7)	5 (27.8)	0 (0.0)	
Study treatment duration in week, grand mean ± S.D.; Median (range, min–max)	12.0 ± 7.1; 12.0 (3.0–24.0)	12.2 ± 7.9; 12.0 (3.0–24.0)	11.6 ± 3.6; 12.0 (6.0–16.0)	0.910
<8 weeks	8 (34.8)	7 (38.9)	1 (20.0)	0.371
8–12 weeks	7 (30.4)	4 (22.2)	3 (60.0)	
>12 weeks	8 (34.8)	7 (38.9)	1 (20.0)	
Funding				
Industry sponsorship	13 (56.5)	13 (72.2)	0 (0.0)	0.001
Partial industry sponsorship	3 (13.0)	3 (16.7)	0 (0.0)	
Academic/government	4 (17.4)	2 (11.1)	2 (40.0)	
None	1 (4.4)	0 (0.0)	1 (20.0)	
Not reported	2 (8.7)	0 (0.0)	2 (40.0)	
Overall risk of bias				
Low	13 (56.5)	12 (66.7)	1 (20.0)	0.127
Some concern	10 (43.5)	6 (33.3)	4 (80.0)	

^†^ On the basis of primary site economy. ^‡^ Interventions were counted independently, as many trials included multiple interventions. Abbreviations: CSU, chronic spontaneous urticaria; S.D., standard deviation.

**Table 3:** Outcome categorization and frequency of outcome reporting.

Outcomes	Number of Trials (%)			*p*-Value
	Overall (*n* = 23)	High-Income Countries (*n* = 18) ^†^	Lower/Upper-Middle-Income Countries (*n* = 5) ^†^	
**Treatment Efficacy (Specific Assessment Tool): Urticaria symptom**				
UAS7 (scale, 0–42)	16 (69.6)	15 (83.3)	1 (20.0)	0.017
Daily UAS (scale, 0–3),	1 (4.4)	0 (0.0)	1 (20.0)	
USS (scale, 0–93),	1 (4.4)	0 (0.0)	1 (20.0)	
VAS (scale, 0–100)	1 (4.4)	1 (5.6)	0 (0.0)	
Not reported	4 (17.4)	2 (11.1)	2 (40.0)	
**Treatment Efficacy (Specific Assessment Tool): Pruritus severity**				
UAS7-subscale itch (scale, 0–21)	13 (56.5)	13 (72.2)	0 (0.0)	0.007
Daily UAS-subscale itch (scale, 0–3)	1 (4.4)	0 (0.0)	1 (20.0)	
VAS (scale, 0–100)	1 (4.4)	1 (5.6)	0 (0.0)	
Not reported	8 (34.8)	4 (22.2)	4 (80.0)	
**Treatment Efficacy (Specific Assessment Tool): Hive severity**				
UAS7-subscale hive/wheal (scale, 0–21)	12 (52.2)	12 (66.7)	0 (0.0)	0.006
Daily UAS-subscale hive/wheal (scale, 0–3)	1 (4.4)	0 (0.0)	1 (20.0)	
Not reported	10 (43.5)	6 (33.3)	4 (80.0)	
**Safety Profile**				
Unacceptability of treatment (all-cause study dropout)	22 (95.6)	18 (100.0)	4 (80.0)	0.217
Occurrence of adverse events reported (participant with ≥1 adverse events)	20 (87.0)	17 (94.4)	3 (60.0)	0.107
Occurrence of serious adverse events reported (participant with ≥1 serious adverse events)	23 (100.0)	18 (100.0)	5 (100.0)	1.000
**Patients-Reported Outcomes (Specific Assessment Tools) ^‡^**				
HRQOL: dermatology—specific measure (DLQI)	10 (43.5)	8 (44.4)	2 (40.0)	1.000
HRQOL: chronic urticaria—specific measure (CU-Q2oL)	4 (17.4)	4 (22.2)	0 (0.0)	0.539
HRQOL: angioedema—specific measure (AE-QoL)	1 (4.4)	1 (5.6)	0 (0.0)	1.000
HRQOL—generic measure (MOSS-SF12)	1 (4.4)	1 (5.6)	0 (0.0)	1.000
Impact on sleep (UPDD-weekly sleep, VAS [scale, 0–100])	1 (4.4)	1 (5.6)	0 (0.0)	1.000
General well-being (WHO-5 well-being index)	5 (21.7)	5 (27.8)	0 (0.0)	0.545

^†^ On the basis of primary site economy. ^‡^ Patient-reported outcomes were counted independently, as many trials included multiple interventions. Abbreviations: AE-QoL, angioedema-quality of life; CU-Q2oL, chronic urticaria-quality of life; DLQI, Dermatology Life Quality Index; HRQOL, health-related quality of life; MOSS-SF12, Medical Outcomes Study Survey—Short Form 12 Item; UAS, urticaria activity score; UAS7, urticaria activity score over 7 days; UPDD, urticaria patient daily diary; USS, urticaria severity score; VAS, visual analog scale; WHO, World Health Organization.

## Data Availability

Not applicable.

## Supplementary Materials

The following supporting information can be downloaded at: https://www.mdpi.com/article/10.3390/ph15101246/s1, Table S1: Systematic review search strategy; Table S2: Grey literature search; Table S3: The PICOTS: study inclusion/exclusion criteria.
