# Supplementary material for: Evidence Gaps in Clinical Trials of Pharmacologic Treatment for H1-Antihistamine-Refractory Chronic Spontaneous Urticaria: A Systematic Review and Future Perspectives
Source: Pharmaceuticals (Basel). 2022 Oct 10;15(10):1246. doi: 10.3390/ph15101246 (PMC9607405; doi:10.3390/ph15101246)
Supplement: Supplementary file 1 [file pharmaceuticals-15-01246-s001.zip › pharmaceuticals-1886699-supplementary.pdf]

Supplementary Materials

Evidence Gaps in Clinical Trials of Pharmacologic Treatment for H1-Antihistamine-Refractory Chronic Spontaneous Urticaria: A Systematic Review and Future Perspectives

Surapon Nochaiwong [1,2,*], Mati Chuamanochan [2,3], Chidchanok Ruengorn [1,2] and Kednapa Thavorn [2,4,5,*]

[1] Department of Pharmaceutical Care, Faculty of Pharmacy, Chiang Mai University, Chiang Mai 50200, Thailand
[2] Pharmacoepidemiology and Statistics Research Center (PESRC), Chiang Mai University, Chiang Mai 50200, Thailand
[3] Division of Dermatology, Department of Internal Medicine, Faculty of Medicine, Chiang Mai University, Chiang Mai 50200, Thailand
[4] Ottawa Hospital Research Institute, Ottawa Hospital, Ottawa, ON K1H 8L6, Canada
[5] School of Epidemiology and Public Health, Faculty of Medicine, University of Ottawa, Ottawa, ON K1G 5Z3, Canada
* Correspondence: surapon.nochaiwong@cmu.ac.th (S.N.); kthavorn@ohri.ca (K.T.); Tel.: +66-53-944-342 (S.N.); +66-613-7378899 (ext. 72330) (K.T.)

Supplementary Materials Content

**Table S1**	Systematic review search strategy	S3
**Table S2**	Grey literature search	S13
**Table S3**	The PICOTS: study inclusion/exclusion criteria	S14

Table S1 Systematic review search strategy: Medline (via OVID), from inception to 19 April, 2021

Search	Query	Items found
#1	exp Urticaria/	16700
#2	Urticaria.mp.	19175
#3	exp Chronic urticaria/	195
#4	Chronic urticaria.mp.	2982
#5	exp Hives/	16700
#6	exp Angioneurotic $edema/	6114
#7	exp Chronic spontaneous urticaria/	195
#8	Chronic spontaneous urticaria.mp.	1042
#9	exp Chronic idiopathic urticaria/	195
#10	Chronic idiopathic urticaria.mp.	762
#11	refractory urticaria.mp.	31
#12	(chronic or persis*) adj (urticaria* or itch* or prurit* or hive* or w$eal* or CU or CSU or CIU).ti,ab,kf.	4271
#13	((refractory or antihistamin* or resistant or inadequate* or unrespons*) adj3 (urticaria* or CU or CSU or CIU)).ti,ab,kf.	632
#14	or/1-13	25282
#15	(anti-immunoglobulin E or anti-IgE* or monoclonal antibodi* or spleen tyrosine kinase inhibitor* or syk inhibitor* or anti-sialic acid binding immunoglobulin-like lectin-8 or anti-Siglec8 or Bruton tyrosine kinase inhibitor* or anti-CRTH2 or CRTH2 antagonist or anti-IL-1 or IL-1 inhibitor* or anti IL-5 or BTK inhibitor* or anti-tumor necrosis factor alpha or anti-TNF* or immunosupp* or calcineurin inhibitor*).ti,ab.	273536
#16	(immunomodulatory or immunotherapy or anti-inflammatory agent* or leukotriene receptor antagonist* or antileukotriene* or LTRA* or antifibrinolytic or anticoagula* or antihistamin* or histamine* antagonist* or up-dosing or updosing or high dosage or increase dose or methylxanthin* or (serotonin inhibitor*) or SSRI* or SNRI* or androgen* or allergen immunotherapy or pseudoallergen-free diet or diet or low histamine diet or probiotic* or herbal therap* or vitamin or nonbiologi* or nonpharmaco* or (autologous adj (blood or serum)) or phototherapy or UV or ultraviolet light or UVA or UVB or PUVA or narrow band UV or NBUVB or plasmapheresis or plasma exchange or acupuncture or alternative treatment* or holistic medicine).ti,ab.	1133592
#17	(omalizumab or ligelizumab or QGE031 or quilizumab or UB-221 or dupilumab or AZD1981 or mepolizumab or reslizumab or ibrutinib or fenebrutinib or GDC-0853 or LOU064 or GSK2646264 or AK002 or adalimumab or abatacept or etanercept or infliximab or benralizumab or rituximab or anakinra or canakinumab or rilonacept).ti,ab.	50210
#18	(cyclosporine or CsA or tacrolimus or sirolimus or methotrexate or cyclophosphamide or azathioprine or mycophenolic acid or mycophenolate mofetil or MMF or MPA or miltefosine or interferon alpha or protease inhibitor* or cromolyn or camostat mesylate or nafamostat mesylate or montelukast or zafirlukast).ti,ab.	251883
#19	(cimetidine or ranitidine or famotidine or vitamin D or vitamin D3 or cholecalciferol or hydroxychloroquine or chloroquine or HCQ or dapsone or sulfasalazine or colchicine or steroid* or immunoglobulin or IVIG or histaglobulin or heparin or warfarin or coumadin or ketotifen or danazol or stanozol or stanozolol or tranexamic acid or dipyridamole or levamisole or doxepin or mirtazapine or theophylline or gold or nifedipine or mizoribine).ti,ab.	820805
#20	or/15-19	2231585
#21	14 and 20	6275
#22	(news or newspaper article or comment or editorial or interview or letter or review or systematic review or case report or case series or cross-sectional).pt.	4991200
#23	21 not 22	4454
#24	exp Clinical Trials as Topic/	355330
#25	randomi$ed controlled trial* or controlled clinical trial*	126823
#26	random allocation or double-blind method or single-blind method or clinical trial/	716445
#27	(controlled clinical trial) or (randomi$ed controlled trial) or (clinical trial).pt	563319
#28	(control* adj2 trial*).tw,kw.	296808
#29	((clinical adj trial*) or (randomly allocated) or (allocated adj2 random*) or randomi$ed or RCT$1 placebo*).tw,kw.	424945
#30	((singl* or doubl* or trebl* or tribl*) adj (blind* or mask* or dumm*)).tw,kw.	178488

#31	(nRCT or nRCTs or non-RCT?).tw,kw.	1046
#32	(control* adj3 ("before and after" or "before after")).tw,kw.	4564
#33	(time series adj3 interrupt*).tw,kw.	3675
#34	(pre- adj3 post-).tw,kw.	90759
#35	(pretest adj3 posttest).tw,kw.	6056
#36	(control* adj2 stud$3).tw,kw.	244925
#37	control group/	1728
#38	(control* adj2 group$1).tw,kw.	533087
#39	or/24-38	2081972
#40	exp comparative study/	1887474
#41	((comparative or comparison) adj stud$3).tw,kw.	114833
#42	exp cohort study/	2115009
#43	(cohort* adj2 stud$3).tw,kw.	256891
#44	exp case control study/	1159190
#45	((case-control* or case-based or case-comparison) adj stud$3).tw,kw.	113193
#46	or/40-45	4035941
#47	23 and 39	844
#48	23 and 46	925
#49	47 or 48	1468
#50	limit 49 to human	1342

Table S1 Systematic review search strategy: Embase (via OVID), from inception to 19 April, 2021 (continued)

Search	Query	Items found
#1	exp Urticaria/	43416
#2	Urticaria.mp.	46085
#3	exp Chronic urticaria/	5638
#4	Chronic urticaria.mp.	6756
#5	exp Hives/	43416
#6	exp Angioneurotic $edema/	20961
#7	exp Chronic spontaneous urticaria/	5638
#8	Chronic spontaneous urticaria.mp.	1775
#9	exp Chronic idiopathic urticaria/	5638
#10	Chronic idiopathic urticaria.mp.	1256
#11	refractory urticaria.mp.	64
#12	(chronic or persis*) adj (urticaria* or itch* or prurit* or hive* or w$eal* or CU or CSU or CIU).ti,ab,kw.	6914
#13	((refractory or antihistamin* or resistant or inadequate* or unrespons*) adj3 (urticaria* or CU or CSU or CIU)).ti,ab,kw.	1156
#14	or/1-13	61991
#15	(anti-immunoglobulin E or anti-IgE* or monoclonal antibodi* or spleen tyrosine kinase inhibitor* or syk inhibitor* or anti-sialic acid binding immunoglobulin-like lectin-8 or anti-Siglec8 or Bruton tyrosine kinase inhibitor* or anti-CRTH2 or CRTH2 antagonist or anti-IL-1 or IL-1 inhibitor* or anti IL-5 or BTK inhibitor* or anti-tumor necrosis factor alpha or anti-TNF* or immunosupp* or calcineurin inhibitor*).ti,ab.	388925
#16	(immunomodulatory or immunotherapy or anti-inflammatory agent* or leukotriene receptor antagonist* or antileukotriene* or LTRA* or antifibrinolytic or anticoagula* or antihistamin* or histamine* antagonist* or up-dosing or updosing or high dosage or increase dose or methylxanthin* or (serotonin inhibitor*) or SSRI* or SNRI* or androgen* or allergen immunotherapy or pseudoallergen-free diet or diet or low histamine diet or probiotic* or herbal therap* or vitamin or nonbiologi* or nonpharmaco* or (autologous adj (blood or serum)) or phototherapy or UV or ultraviolet light or UVA or UVB or PUVA or narrow band UV or NBUVB or plasmapheresis or plasma exchange or acupuncture or alternative treatment* or holistic medicine).ti,ab.	1477108
#17	(omalizumab or ligelizumab or QGE031 or quilizumab or UB-221 or dupilumab or AZD1981 or mepolizumab or reslizumab or ibrutinib or fenebrutinib or GDC-0853 or LOU064 or GSK2646264 or AK002 or adalimumab or abatacept or etanercept or infliximab or benralizumab or rituximab or anakinra or canakinumab or rilonacept).ti,ab.	109824
#18	(cyclosporine or CsA or tacrolimus or sirolimus or methotrexate or cyclophosphamide or azathioprine or mycophenolic acid or mycophenolate mofetil or MMF or MPA or miltefosine or interferon alpha or protease inhibitor* or cromolyn or camostat mesylate or nafamostat mesylate or montelukast or zafirlukast).ti,ab.	361710
#19	(cimetidine or ranitidine or famotidine or vitamin D or vitamin D3 or cholecalciferol or hydroxychloroquine or chloroquine or HCQ or dapsone or sulfasalazine or colchicine or steroid* or immunoglobulin or IVIG or histaglobulin or heparin or warfarin or coumadin or ketotifen or danazol or stanozol or stanozolol or tranexamic acid or dipyridamole or levamisole or doxepin or mirtazapine or theophylline or gold or nifedipine or mizoribine).ti,ab.	1064755
#20	or/15-19	2907075
#21	14 and 20	18460
#22	(news or newspaper article or comment or editorial or interview or letter or review or systematic review or case report or case series or cross-sectional).pt.	4564090
#23	21 not 22	14580
#24	exp Clinical Trials as Topic/	350405
#25	randomi$ed controlled trial* or controlled clinical trial*	506345
#26	random allocation or double-blind method or single-blind method or clinical trial/	1022623
#27	(controlled clinical trial) or (randomi$ed controlled trial) or (clinical trial).pt	493863
#28	(control* adj2 trial*).tw,kw.	402912
#29	((clinical adj trial*) or (randomly allocated) or (allocated adj2 random*) or randomi$ed or RCT$1 placebo*).tw,kw.	623493

#30	((singl* or doubl* or trebl* or tribl*) adj (blind* or mask* or dumm*)).tw,kw.	244888
#31	(nRCT or nRCTs or non-RCT?).tw,kw.	1464
#32	(control* adj3 ("before and after" or "before after")).tw,kw.	5935
#33	(time series adj3 interrupt*).tw,kw.	4720
#34	(pre- adj3 post-).tw,kw.	163587
#35	(pretest adj3 posttest).tw,kw.	7322
#36	(control* adj2 stud$3).tw,kw.	330935
#37	control group/	110007
#38	(control* adj2 group$1).tw,kw.	767571
#39	or/24-38	2973357
#40	exp comparative study/	1364497
#41	((comparative or comparison) adj stud$3).tw,kw.	131808
#42	exp cohort study/	685417
#43	(cohort* adj2 stud$3).tw,kw.	390674
#44	exp case control study/	187146
#45	((case-control* or case-based or case-comparison) adj stud$3).tw,kw.	149853
#46	or/40-45	2338613
#47	23 and 39	3199
#48	23 and 46	1062
#49	47 or 48	3802
#50	limit 49 to human	3614

Table S1 Systematic review search strategy: PubMed, from inception to 19 April, 2021 (continued)

Search	Query	Items found
#1	(((((((((((urticaria[MeSH Terms]) OR (urticaria[Title/Abstract])) OR (chronic urticaria)) OR (hives)) OR (angioneurotic $edema)) OR (chronic spontaneous urticaria)) OR (chronic idiopathic urticaria)) OR (refractory urticaria)) OR (antihistamin* resistant chronic urticaria)) OR (CU)) OR (CSU)) OR (CIU)	165414
#2	(((((((((((((((((anti-immunoglobulin E[Title/Abstract]) OR (anti-IgE*[Title/Abstract])) OR (monoclonal antibodi*[Title/Abstract])) OR (spleen tyrosine kinase inhibitor*[Title/Abstract])) OR (syk inhibitor*[Title/Abstract])) OR (anti-sialic acid binding immunoglobulin-like lectin-8[Title/Abstract])) OR (anti-Siglec-8[Title/Abstract])) OR (Bruton tyrosine kinase inhibitor*[Title/Abstract])) OR (anti-CRTH2[Title/Abstract])) OR (CRTH2 antagonist[Title/Abstract])) OR (anti-IL-1[Title/Abstract])) OR (IL-1 inhibitor*[Title/Abstract])) OR (anti IL-5[Title/Abstract])) OR (BTK inhibitor*[Title/Abstract])) OR (anti-tumor necrosis factor alpha[Title/Abstract])) OR (anti-TNF*[Title/Abstract])) OR (immunosupp*[Title/Abstract])) OR (calcineurin inhibitor*[Title/Abstract])	279297
#3	(((((((((((((((((((((((((((((((((((((((immunomodulatory[Title/Abstract]) OR (immunotherapy[Title/Abstract])) OR (anti-inflammatory agent*[Title/Abstract])) OR (leukotriene receptor antagonist*[Title/Abstract])) OR (anti-leukotriene*[Title/Abstract])) OR (LTRA*[Title/Abstract])) OR (anti-fibrinolytic[Title/Abstract])) OR (anticoagula*[Title/Abstract])) OR (antihistamin*[Title/Abstract])) OR (histamine* antagonist*[Title/Abstract])) OR (up-dosing[Title/Abstract])) OR (high dosage[Title/Abstract])) OR (increase dose[Title/Abstract])) OR (methylxanthin*[Title/Abstract])) OR (serotonin inhibitor*[Title/Abstract])) OR (SSRI*[Title/Abstract])) OR (SNRI*[Title/Abstract])) OR (androgen*[Title/Abstract])) OR (allergen immunotherapy[Title/Abstract])) OR (pseudoallergen-free diet[Title/Abstract])) OR (diet[Title/Abstract])) OR (low histamine diet[Title/Abstract])) OR (probiotic*[Title/Abstract])) OR (herbal therap*[Title/Abstract])) OR (vitamin[Title/Abstract])) OR (nonbiologi*[Title/Abstract])) OR (nonpharmaco*[Title/Abstract])) OR (autologous adj (blood[Title/Abstract] OR serum)[Title/Abstract])) OR (phototherapy[Title/Abstract])) OR (UV[Title/Abstract])) OR (ultraviolet light[Title/Abstract])) OR (UVA[Title/Abstract])) OR (UVB[Title/Abstract])) OR (PUVA[Title/Abstract])) OR (narrow band UV[Title/Abstract])) OR (NBUVB[Title/Abstract])) OR (plasmapheresis[Title/Abstract])) OR (plasma exchange[Title/Abstract])) OR (acupuncture[Title/Abstract])) OR (alternative treatment*[Title/Abstract])) OR (holistic medicine[Title/Abstract])	1181118
#4	(((((((((((((((((((((((omalizumab[Title/Abstract]) OR (ligelizumab[Title/Abstract])) OR (QGE031[Title/Abstract])) OR (quilizumab[Title/Abstract])) OR (UB-221[Title/Abstract])) OR (dupilumab[Title/Abstract])) OR (AZD1981[Title/Abstract])) OR (mepolizumab[Title/Abstract])) OR (reslizumab[Title/Abstract])) OR (ibrutinib[Title/Abstract])) OR (fenebrutinib[Title/Abstract])) OR (GDC-0853[Title/Abstract])) OR (LOU064[Title/Abstract])) OR (GSK2646264[Title/Abstract])) OR (AK002[Title/Abstract])) OR (adalimumab[Title/Abstract])) OR (abatacept[Title/Abstract])) OR (etanercept[Title/Abstract])) OR (infliximab[Title/Abstract])) OR (benralizumab[Title/Abstract])) OR (rituximab[Title/Abstract])) OR (anakinra[Title/Abstract])) OR (canakinumab[Title/Abstract])) OR (rilonacept[Title/Abstract])	51386
#5	((((((((((((((((((cyclosporine[Title/Abstract]) OR (CsA[Title/Abstract])) OR (tacrolimus[Title/Abstract])) OR (sirolimus[Title/Abstract])) OR (methotrexate[Title/Abstract])) OR (cyclophosphamide[Title/Abstract])) OR (azathioprine[Title/Abstract])) OR (mycophenolic acid[Title/Abstract])) OR (mycophenolate mofetil[Title/Abstract])) OR (MMF[Title/Abstract])) OR (MPA[Title/Abstract])) OR (miltefosine[Title/Abstract])) OR (interferon alpha[Title/Abstract])) OR (protease inhibitor*[Title/Abstract])) OR (cromolyn[Title/Abstract])) OR (camostat mesylate[Title/Abstract])) OR (nafamostat mesylate[Title/Abstract])) OR (montelukast[Title/Abstract])) OR (zafirlukast[Title/Abstract])	256411
#6	(((((((((((((((((((((((((((((((cimetidine[Title/Abstract]) OR (ranitidine[Title/Abstract])) OR (famotidine[Title/Abstract])) OR (vitamin D[Title/Abstract])) OR (vitamin D3[Title/Abstract])) OR (cholecalciferol[Title/Abstract])) OR (hydroxychloroquine[Title/Abstract])) OR (chloroquine[Title/Abstract])) OR (HCQ[Title/Abstract])) OR (dapsone[Title/Abstract])) OR (sulfasalazine[Title/Abstract])) OR (colchicine[Title/Abstract])) OR (steroid*[Title/Abstract])) OR (immunoglobulin[Title/Abstract])) OR (IVIG[Title/Abstract])) OR (histaglobulin[Title/Abstract])) OR (heparin[Title/Abstract])) OR (warfarin[Title/Abstract]))	837578

	OR (coumadin[Title/Abstract])) OR (ketotifen[Title/Abstract])) OR (danazol[Title/Abstract])) OR (stanozol[Title/Abstract])) OR (stanozolol[Title/Abstract])) OR (tranexamic acid[Title/Abstract])) OR (dipyridamole[Title/Abstract])) OR (levamisole[Title/Abstract])) OR (doxepin[Title/Abstract])) OR (mirtazapine[Title/Abstract])) OR (theophylline[Title/Abstract])) OR (gold[Title/Abstract])) OR (nifedipine[Title/Abstract])) OR (mizoribine[Title/Abstract])	
#7	#2 OR #3 OR #4 OR #5 OR #6	2295876
#8	#1 AND #7	20161
#9	(((((((Case Reports[Publication Type]) OR Comment[Publication Type]) OR Editorial[Publication Type]) OR Guideline[Publication Type]) OR Letter[Publication Type]) OR News[Publication Type]) OR Newspaper Article[Publication Type]) OR Review[Publication Type]	6760906
#10	#8 NOT #9	16190
#11	Filters: Humans	5630

Table S1 Systematic review search strategy: Cochrane Library, from inception to 19 April, 2021 (continued)

Search	Query	Items found
#1	MeSH descriptor: [Chronic Urticaria] explode all trees	7
#2	(Urticarial OR “chronic urticarial” OR hives OR “angioneurotic $edema” OR “chronic spontaneous urticarial” OR “chronic idiopathic urticarial” OR “refractory urticarial” OR “antihistamin* resistant chronic urticarial” OR CSU OR CIU):ti,ab,kw	1281
#3	#1 OR #2	1286
#4	(“anti-immunoglobulin” OR anti-IgE* OR “monoclonal antibodi*” OR “spleen tyrosine kinase inhibitor*” OR “syk inhibitor*” OR “anti-sialic acid binding immunoglobulin-like lectin-8” OR “anti-Siglec-8” OR “Bruton tyrosine kinase inhibitor*” OR “anti-CRTH2” OR “CRTH2 antagonist” OR anti-IL-1 OR “IL-1 inhibitor*” OR anti IL-5 OR “BTK inhibitor*” OR “anti-tumor necrosis factor alpha” OR anti-TNF* OR immunosupp* OR “calcineurin inhibitor*”):ti,ab,kw	17775
#5	(immunomodulatory OR immunotherapy OR “anti-inflammatory agent*” OR “leukotriene receptor antagonist*” OR anti-leukotriene* OR LTRA* OR anti-fibrinolytic OR anticoagula* OR antihistamin* OR “histamine* antagonist*” OR up-dosing OR “high dosage” OR “increase dose” OR methylxanthin* OR “serotonin inhibitor*” OR SSRI* OR SNRI* OR androgen* OR “allergen immunotherapy” OR “pseudoallergen-free diet” OR diet OR “low histamine diet” OR probiotic* OR “herbal therap*” OR vitamin OR nonbiologi* OR nonpharmaco* OR autologous blood OR autologous serum OR phototherapy OR UV OR “ultraviolet light” OR UVA OR UVB OR PUVA OR “narrow band UV” OR NBUVB OR plasmapheresis OR “plasma exchange” OR acupuncture OR “alternative treatment*” OR “holistic medicine”):ti,ab,kw	169394
#6	(omalizumab OR ligelizumab OR QGE031 OR quilizumab OR UB-221 OR dupilumab OR AZD1981 OR mepolizumab OR reslizumab OR ibrutinib OR fenebrutinib OR GDC-0853 OR LOU064 OR GSK2646264 OR AK002 OR adalimumab OR abatacept OR etanercept OR infliximab OR benralizumab OR rituximab OR anakinra OR canakinumab OR rilonacept):ti,ab,kw	15168
#7	(cyclosporine OR CsA OR tacrolimus OR sirolimus OR methotrexate OR cyclophosphamide OR azathioprine OR mycophenolic acid OR mycophenolate mofetil OR MMF OR MPA OR miltefosine OR interferon alpha OR protease inhibitor* OR cromolyn OR camostat mesylate OR nafamostat mesylate OR montelukast OR zafirlukast):ti,ab,kw	51373
#8	(cimetidine OR ranitidine OR famotidine OR vitamin D OR vitamin D3 OR cholecalciferol OR hydroxychloroquine OR chloroquine OR HCQ OR dapsone OR sulfasalazine OR colchicine OR steroid* OR immunoglobulin OR IVIG OR histaglobulin OR heparin OR warfarin OR coumadin OR ketotifen OR danazol OR stanozol OR stanozolol OR “tranexamic acid” OR dipyridamole OR levamisole OR doxepin OR mirtazapine OR theophylline OR gold OR nifedipine OR mizoribine):ti,ab,kw	107716
#9	#4 OR #5 OR #6 OR #7 OR #8	300602
#10	#3 AND #9	619
#11	limit in trials	594

Table S1 Systematic review search strategy: Web of Science, from inception to 19 April, 2021 (continued)

Search	Query	Items found
#1	TS=(urticarial OR "chronic urticarial" OR hives OR "angioneurotic $edema" OR "chronic spontaneous urticarial" OR "chronic idiopathic urticarial" OR "refractory urticarial" OR "antihistamin* resistant chronic urticarial" OR CSU OR CIU)	14451
#2	TS=("anti-immunoglobulin" OR anti-IgE* OR "monoclonal antibodi*" OR "spleen tyrosine kinase inhibitor*" OR "syk inhibitor*" OR "anti-sialic acid binding immunoglobulin-like lectin-8" OR "anti-Siglec-8" OR "Bruton tyrosine kinase inhibitor*" OR "anti-CRTH2" OR "CRTH2 antagonist" OR anti-IL-1 OR "IL-1 inhibitor*" OR anti IL-5 OR "BTK inhibitor*" OR "anti-tumor necrosis factor alpha" OR anti-TNF* OR immunosupp* OR "calcineurin inhibitor*")	362714
#3	TS=(immunomodulatory OR immunotherapy OR "anti-inflammatory agent*" OR "leukotriene receptor antagonist*" OR anti-leukotriene* OR LTRA* OR anti-fibrinolytic OR anticoagula* OR antihistamin* OR "histamine* antagonist*" OR up-dosing OR "high dosage" OR "increase dose" OR methylxanthin* OR "serotonin inhibitor*" OR SSRI* OR SNRI* OR androgen* OR "allergen immunotherapy" OR "pseudoallergen-free diet" OR diet OR "low histamine diet" OR probiotic* OR "herbal therap*" OR vitamin OR nonbiologi* OR nonpharmaco* OR autologous blood OR autologous serum OR phototherapy OR UV OR "ultraviolet light" OR UVA OR UVB OR PUVA OR "narrow band UV" OR NBUVB OR plasmapheresis OR "plasma exchange" OR acupuncture OR "alternative treatment*" OR "holistic medicine")	2046063
#4	TS=(omalizumab OR ligelizumab OR QGE031 OR quilizumab OR UB-221 OR dupilumab OR AZD1981 OR mepolizumab OR reslizumab OR ibrutinib OR fenebrutinib OR GDC-0853 OR LOU064 OR GSK2646264 OR AK002 OR adalimumab OR abatacept OR etanercept OR infliximab OR benralizumab OR rituximab OR anakinra OR canakinumab OR rilonacept)	114884
#5	TS=(cyclosporine OR CsA OR tacrolimus OR sirolimus OR methotrexate OR cyclophosphamide OR azathioprine OR mycophenolic acid OR mycophenolate mofetil OR MMF OR MPA OR miltefosine OR interferon alpha OR protease inhibitor* OR cromolyn OR camostat mesylate OR nafamostat mesylate OR montelukast OR zafirlukast)	582718
#6	TS=(cimetidine OR ranitidine OR famotidine OR vitamin D OR vitamin D3 OR cholecalciferol OR hydroxychloroquine OR chloroquine OR HCQ OR dapsone OR sulfasalazine OR colchicine OR steroid* OR immunoglobulin OR IVIG OR histaglobulin OR heparin OR warfarin OR coumadin OR ketotifen OR danazol OR stanozol OR stanozolol OR "tranexamic acid" OR dipyridamole OR levamisole OR doxepin OR mirtazapine OR theophylline OR gold OR nifedipine OR mizoribine)	1416098
#7	#2 OR #3 OR #4 OR #5 OR #6	3744523
#8	#1 AND #7	4728
#9	Refined by: [excluding] DOCUMENT TYPES: (CASE REPORT OR BOOK OR LETTER OR EDITORIAL MATERIAL OR NEWS OR REVIEW)	3332

Table S1 Systematic review search strategy: Scopus, from inception to 19 April, 2021 (continued)

Search	Query	Items found
#1	TITLE-ABS-KEY (urticarial OR "chronic urticarial" OR hives OR "angioneurotic $edema" OR "chronic spontaneous urticarial" OR "chronic idiopathic urticarial" OR "refractory urticarial" OR "antihistamin* resistant chronic urticarial" OR csu OR ciu)	29533
#2	TITLE-ABS-KEY ("anti-immunoglobulin" OR anti-ige* OR "monoclonal antibodi*" OR "spleen tyrosine kinase inhibitor*" OR "syk inhibitor*" OR "anti-sialic acid binding immunoglobulin-like lectin-8" OR "anti-Siglec-8" OR "Bruton tyrosine kinase inhibitor*" OR "anti-CRTH2" OR "CRTH2 antagonist" OR anti-il-1 OR "IL-1 inhibitor*" OR anti AND il-5 OR "BTK inhibitor*" OR "anti-tumor necrosis factor alpha" OR anti-tnf* OR immunosupp* OR "calcineurin inhibitor*")	72306
#3	TITLE-ABS-KEY (immunomodulatory OR immunotherapy OR "anti-inflammatory agent*" OR "leukotriene receptor antagonist*" OR anti-leukotriene* OR ltra* OR anti-fibrinolytic OR anticoagula* OR antihistamin* OR "histamine* antagonist*" OR up-dosing OR "high dosage" OR "increase dose" OR methylxanthin* OR "serotonin inhibitor*" OR ssri* OR snri* OR androgen* OR "allergen immunotherapy" OR "pseudoallergen-free diet" OR diet OR "low histamine diet" OR probiotic* OR "herbal therap*" OR vitamin OR nonbiologi* OR nonpharmaco* OR autologous AND blood OR autologous AND serum OR phototherapy OR uv OR "ultraviolet light" OR uva OR uvb OR puva OR "narrow band UV" OR nbuvb OR plasmapheresis OR "plasma exchange" OR acupuncture OR "alternative treatment*" OR "holistic medicine")	143838
#4	TITLE-ABS-KEY (omalizumab OR ligelizumab OR qge031 OR quilizumab OR ub-221 OR dupilumab OR azd1981 OR mepolizumab OR reslizumab OR ibrutinib OR fenebrutinib OR gdc-0853 OR lou064 OR gsk2646264 OR ak002 OR adalimumab OR abatacept OR etanercept OR infliximab OR benralizumab OR rituximab OR anakinra OR canakinumab OR rilonacept)	129890
#5	TITLE-ABS-KEY (cyclosporine OR csa OR tacrolimus OR sirolimus OR methotrexate OR cyclophosphamide OR azathioprine OR "mycophenolic acid" OR "mycophenolate mofetil" OR mmf OR mpa OR miltefosine OR "interferon alpha" OR "protease inhibitor*" OR cromolyn OR "camostat mesylate" OR "nafamostat mesylate" OR montelukast OR zafirlukast)	860087
#6	TITLE-ABS-KEY (cimetidine OR ranitidine OR famotidine OR "vitamin D" OR "vitamin D3" OR cholecalciferol OR hydroxychloroquine OR chloroquine OR hcq OR dapsone OR sulfasalazine OR colchicine OR steroid* OR immunoglobulin OR ivig OR histaglobulin OR heparin OR warfarin OR coumadin OR ketotifen OR danazol OR stanozol OR stanozolol OR "tranexamic acid" OR dipyridamole OR levamisole OR doxepin OR mirtazapine OR theophylline OR gold OR nifedipine OR mizoribine)	2277803
#7	#2 OR #3 OR #4 OR #5 OR #6	3159228
#8	#1 AND #7	9400
#9	Filters: (EXCLUDE (DOCTYPE , "re") OR EXCLUDE (DOCTYPE , "le") OR EXCLUDE (DOCTYPE , "no") OR EXCLUDE (DOCTYPE , "sh") OR EXCLUDE (DOCTYPE , "ed") OR EXCLUDE (DOCTYPE , "ch") OR EXCLUDE (DOCTYPE , "cr") OR EXCLUDE (DOCTYPE , "tb")) AND (EXCLUDE (SRCTYPE , "k") OR EXCLUDE (SRCTYPE , "b") OR EXCLUDE (SRCTYPE , "d"))	6042

Table S1 Systematic review search strategy: CINAHL, from inception to 19 April, 2021 (continued)

Search	Query	Items found
#1	AB (urticarial OR "chronic urticarial" OR hives OR "angioneurotic $edema" OR "chronic spontaneous urticarial" OR "chronic idiopathic urticarial" OR "refractory urticarial" OR "antihistamin* resistant chronic urticarial" OR csu OR ciu)	773
#2	AB ("anti-immunoglobulin" OR anti-ige* OR "monoclonal antibodi*" OR "spleen tyrosine kinase inhibitor*" OR "syk inhibitor*" OR "anti-sialic acid binding immunoglobulin-like lectin-8" OR "anti-Siglec-8" OR "Bruton tyrosine kinase inhibitor*" OR "anti-CRTH2" OR "CRTH2 antagonist" OR anti-il-1 OR "IL-1 inhibitor*" OR anti AND il-5 OR "BTK inhibitor*" OR "anti-tumor necrosis factor alpha" OR anti-tnf* OR immunosupp* OR "calcineurin inhibitor*")	20762
#3	AB (immunomodulatory OR immunotherapy OR "anti-inflammatory agent*" OR "leukotriene receptor antagonist*" OR anti-leukotriene* OR ltra* OR anti-fibrinolytic OR anticoagula* OR antihistamin* OR "histamine* antagonist*" OR up-dosing OR "high dosage" OR "increase dose" OR methylxanthin* OR "serotonin inhibitor*" OR ssri* OR snri* OR androgen* OR "allergen immunotherapy" OR "pseudoallergen-free diet" OR diet OR "low histamine diet" OR probiotic* OR "herbal therap*" OR vitamin OR nonbiologi* OR nonpharmaco* OR autologous AND blood OR autologous AND serum OR phototherapy OR uv OR "ultraviolet light" OR uva OR uvb OR puva OR "narrow band UV" OR nbuvb OR plasmapheresis OR "plasma exchange" OR acupuncture OR "alternative treatment*" OR "holistic medicine")	166285
#4	AB (omalizumab OR ligelizumab OR qge031 OR quilizumab OR ub-221 OR dupilumab OR azd1981 OR mepolizumab OR reslizumab OR ibrutinib OR fenebrutinib OR gdc-0853 OR lou064 OR gsk2646264 OR ak002 OR adalimumab OR abatacept OR etanercept OR infliximab OR benralizumab OR rituximab OR anakinra OR canakinumab OR rilonacept)	9508
#5	AB (cyclosporine OR csa OR tacrolimus OR sirolimus OR methotrexate OR cyclophosphamide OR azathioprine OR "mycophenolic acid" OR "mycophenolate mofetil" OR mmf OR mpa OR miltefosine OR "interferon alpha" OR "protease inhibitor*" OR cromolyn OR "camostat mesylate" OR "nafamostat mesylate" OR montelukast OR zafirlukast)	24091
#6	AB (cimetidine OR ranitidine OR famotidine OR "vitamin D" OR "vitamin D3" OR cholecalciferol OR hydroxychloroquine OR chloroquine OR hcq OR dapsone OR sulfasalazine OR colchicine OR steroid* OR immunoglobulin OR ivig OR histaglobulin OR heparin OR warfarin OR coumadin OR ketotifen OR danazol OR stanozol OR stanozolol OR "tranexamic acid" OR dipyridamole OR levamisole OR doxepin OR mirtazapine OR theophylline OR gold OR nifedipine OR mizoribine)	88071
#7	S2 OR S3 OR S4 OR S5 OR S6	266069
#8	S1 AND S7	224
#9	Expanders - Apply equivalent subjects<br>Source Types - Academic Journals	210

Table S2 Grey literature search

Ongoing clinical trial register

- Australia and New Zealand's (ANZCTR) (http://www.anzctr.org.au)
- Brazilian Clinical Trials Registry (ReBec) (http://www.ensaiosclinicos.gov.br)
- Chinese Clinical Trial Registry (ChiCTR) (http://www.chictr.org.cn)
- Clinical Research Information Service (CRiS), Republic of Korea (http://cris.cdc.go.kr)
- Clinical Trials Registry - India (CTRI) (http://ctri.nic.in)
- Cuban Public Registry of Clinical Trials(RPCEC) (http://registroclinico.sld.cu)
- EU Clinical Trials Register (EU-CTR) (https://www.clinicaltrialsregister.eu)
- German Clinical Trials Register (DRKS) (http://www.drks.de)
- Iranian Registry of Clinical Trials (IRCT) (http://www.irct.ir)
- Japan Primary Registries Network (https://rctportal.niph.go.jp)
- The Netherlands Trial Register (http://www.trialregister.nl)
- Pan African Clinical Trial Registry (PACTR) (http://www.pactr.org)
- Peruvian Registry of Clinical Trials (http://www.ins.gob.pe/ensayosclinicos)
- Philippine Health Research Registry (http://registry.healthresearch.ph)
- Sri Lanka Clinical Trials Registry (SLCTR) (http://www.slctr.lk)
- South African National Clinical Trials Register (http://www.sanctr.gov.za)
- Swiss FOPH Human Research Projects (https://www.kofam.ch/en/swiss-clinical-trials-portal.html)
- Tanzania Clinical Trial Registry (http://www.tzctr.or.tz)
- Thai Clinical Trials Registry (http://www.clinicaltrials.in.th)
- The United Kingdoms' ISRCTN registry (http://www.isrctn.com)
- The US National Institutes of Health Ongoing Trials Registry (http://clinicaltrials.gov)
- The World Health Organization International Clinical Trials Registry Platform (ICTRP) (https://www.who.int/ictrp)

Preprint databases

- medRxiv (https://www.medrxiv.org)
- bioRxiv (https://www.biorxiv.org)
- Research Square (https://www.researchsquare.com)

Table S3 The PICOTS: study inclusion/exclusion criteria

Category	Criteria for inclusion	Criteria for exclusion
Populations	• Adolescents or adults (12 years or older) diagnosed with refractory to H1-antihistamines (standard dose or up-dosing) CSU	• Studies that recruiting participants aged less than 12 years or unclear definition of CSU<br>• In vitro or animal studies
Interventions	• Pharmacological treatment with any type of administered treatments for refractory to H1-antihistamine CSU	• Studies that using non-pharmacological treatments<br>• Studies with the disconnected node of treatments
Comparators	• Placebo, active comparator, or standard of care	• Studies without control groups (single arm studies)
Outcomes	• Primary outcomes<br>o Treatment response: change in urticarial symptoms score using validated measurement tools<br>o Acceptability of treatment (all-cause discontinuation)<br>o Patient-reported QOL: general dermatology-specific and chronic urticaria-specific measures<br>• Secondary outcomes<br>o Treatment response: change in pruritus severity score using validated measurement tools<br>o Treatment response: change in hives (wheal) severity score using validated measurement tools<br>o Incidence of adverse event (patient with at least one reported adverse event)<br>o Incidence of serious adverse event (patients with at least one reported serious adverse event)<br>o Angioedema-related QOL<br>o Impact on sleep<br>o Symptom burden: fatigue, dyspnea, pain, difficulty in eating or swallowing<br>o Work impairment: absenteeism, presenteeism, work productivity and/or school performance	• Studies not providing data for calculate the efficacy or safety of primary outcomes<br>• Studies with a follow-up period of less than two weeks

	o Psychosocial aspects: depressive symptoms, anxiety, distress, and well-being)<br>o Healthcare utilization: emergency/unplanned visit during follow-up and costs of care	
Timing	• An extensive search strategy from the inception of bibliographic databases forward to assure all published literature was identified	• No limit timing of start date<br>• Studies were not limited by language
Setting	• Experimental study: RCTs (parallel or crossover trials)	• Cohort, case-control, cross-sectional, N-of-one, case series/case reports, reviews, and systematic review and meta-analysis

Abbreviations: CSU, chronic spontaneous urticarial; QOL, quality of life; RCTs, randomized controlled trials.
